# Prognostic nutritional index negative associated with mortality in older Japanese patients with dysphagia

**DOI:** 10.3389/fnut.2025.1586248

**Published:** 2025-05-08

**Authors:** Ruijun Pei, Donghao Wang

**Affiliations:** Department of Intensive Care Unit, Tianjin Medical University Cancer Institute and Hospital, National Clinical Research Center for Cancer, Key Laboratory of Cancer Prevention and Therapy, Tianjin, Tianjin's Clinical Research Center for Cancer, Tianjin, China

**Keywords:** dysphagia, mortality, albumin, total lymphocyte count, prognostic nutritional index

## Abstract

**Background:**

The prognostic nutritional index (PNI) has been proven to represent a biomarker for predicting prognosis in many groups of patients with severe diseases. However, few studies have investigated the association between PNI and mortality in Japan older people with dysphagia patients.

**Objective:**

This retrospective cohort study aimed to assess the prognostic value of PNI in older Japanese patients with dysphagia.

**Methods:**

We analyzed data from 248 patients diagnosed with dysphagia at a single center between January 2014 and January 2017. According to PNI score, all patients were divided into normal nutrition group (PNI ≥ 38), moderate malnutrition group (35 ≤ PNI < 38) and severe malnutrition group (PNI < 35). Cox regression analysis was used to compare the mortality rates among the three groups. Subgroup analyses were conducted, and Kaplan-Meier curves were used to determine the median survival times.

**Results:**

The mean age of the patients was 83.0 ± 9.3 years, with a male-to-female ratio of 0.64:1. Of the patients, 180 received percutaneous endoscopic gastrostomy (PEG) and 68 received total parenteral nutrition (TPN). After adjusting for all covariates, the multivariable Cox regression analysis revealed a significant association between PNI and the risk of mortality (HR = 0.94, 95% CI: 0.92–0.97, *P* < 0.001). Compared with the normal nutrition group, the mortality rate of severe malnutrition group was significantly higher (*P* = 0.007). The adjusted hazard ratios for the severe and moderate malnutrition groups were 1.83 (95%CI: 1.18–2.84, *P* = 0.007) and 1.39 (95%CI: 0.81–2.4, *P* = 0.234), respectively. Kaplan-Meier curves indicated median survival times of 189, 447, and 864 days for severe malnutrition group, moderate malnutrition group, and normal nutrition group, respectively.

**Conclusion:**

PNI was negatively associated with mortality in older Japanese patients with dysphagia. There was no interaction for the subgroup analysis. The result was stable.

## 1 Introduction

Dysphagia, a prevalent condition among older adults, has been found in 10%−27% of people in the community, with a prevalence of up to 50% of care home residents and elderly hospitalized patients (1). Dysphagia is frequently observed among stroke patients, with its prevalence varying from 37% to 78% (2). It is more prevalent among the elderly diagnosed with neurological disorders [the incidence of dysphagia is approximately 80% in patients with Alzheimer's disease and around 60% in those with Parkinson's disease (3)]. Patients diagnosed with dysphagia may encounter malnutrition, pneumonia, and dehydration, which subsequently contribute to an elevation of both long-term hospitalization rates and mortality. In a large cross-sectional study targeting nursing home residents, it was revealed that the 6-month mortality of residents experiencing dysphagia was 24.7%, compared with 11.9% in those without dysphagia (*P* < 0.001) (4). In the hospital setting, patients diagnosed with dysphagia exhibited a 1.7-times (95%CI: 1.67 to 1.74 times) (5) in the likelihood of mortality when compared to those without dysphagia. Moreover, dysphagia is correlated with a longer hospital length of stay (LOS). A study based on a large sample database of hospitalized patients in the United States from 2009 to 2013 demonstrated that the mean hospital LOS for patients with dysphagia was 3.8 days longer than that for those without dysphagia (8.8 days vs. 5.0 days; *P* < 0.001) (5). The average total hospitalization cost for patients with dysphagia increased by $6,243 ($19,244 vs. $13,001; *P* < 0.001) (5). Consequently, it is of crucial importance to detect dysphagia at an early stage and conduct active interventions to prevent patients from suffering more severe consequences.

The Prognostic Nutritional Index (PNI) was first proposed in 1984, calculated by combining serum albumin (ALB) levels and total lymphocyte count (6). Initially, it was used to evaluate the preoperative surgical risks of digestive system tumors (7). In recent years, it has been demonstrated to possess favorable predictive value across a diverse range of diseases, especially in chronic diseases and malignant tumors (8–10). Lymphocytes play a crucial role in the host immune response. Lymphocytopenia indicates a decline in the ability to resist infections. ALB, an acute-phase protein with a long half-life, is regarded as an indicator of nutritional status and inflammatory activity. A low ALB level suggests impaired capacity for protein synthesis and a malnourished state. Overall, a low PNI indicates hypoalbuminemia and lymphocytopenia, reflecting the nutritional status and overall health status of patients, which is associated with increased mortality and poor prognosis (11).

However, a comprehensive guideline for the management of dysphagia in the elderly is still lacking. Early assessment and prediction of the adverse prognosis associated with dysphagia are crucial. While previous studies have identified the prognostic value of PNI in various diseases, the research evidence regarding the correlation between PNI and mortality in patients with dysphagia is limited. Factors predicting mortality in patients with dysphagia remain under investigation. Therefore, the present study aims to investigate whether PNI is independently associated with mortality in elderly patients with dysphagia residing in Japan.

## 2 Materials and methods

### 2.1 Data source

The data utilized in this study were obtained from the Dryad Digital Repository (12), which represents a platform affording users unfettered access to and retrieval of the original data. All authors have waived copyright to the original research data. Therefore, these data were used for secondary analysis without infringing on the authors' rights. Since all the data used were extracted from online data resources, no institutional ethics committee approval was required.

### 2.2 Study design

This retrospective cohort study was carried out at a single center, with a particular focus on older patients afflicted with dysphagia and who underwent percutaneous endoscopic gastrostomy (PEG) or total parenteral nutrition (TPN), including implantable central venous ports (PORT), non-tunneled central venous catheters (NT-CVC), and peripherally inserted central catheters (PICC) during the period from January 2014 to January 2017. In this dataset, the evaluation of dysphagia was performed clinically by a doctor, a nurse, and a speech-language pathologist, in conjunction with assessments via video fluoroscopy. Subsequently, it was clearly demonstrated that each patient exhibited severe dysphagia. Patients with terminal cancer, those necessitating PEG for gastric decompression, and individuals who had undergone PEG prior to January 2014 were excluded from the present study.

The following clinical information was collected and processed for secondary analysis, including age, gender, underlying diseases such as cerebrovascular disease, severe dementia, neuromuscular diseases, aspiration pneumonia, ischemic heart disease (IHD), chronic heart failure, chronic lung disease, chronic liver disease, chronic kidney disease (CKD), the presence of PORT, NT-CVC, PICC, PEG, oral intake recovery, blood test results, body mass index, daily calorie intake, discharge to home, severe pneumonia, and sepsis, etc. The blood test results were performed within 7 days before the start of PEG feeding or TPN (12). In addition, the survival status and follow-up duration of each patient with dysphagia were also determined. Grouping was carried out according to PNI, with PNI > 38 reflecting the normal nutritional status group, 35–38 as the moderate malnutrition group, and < 35 as the severe malnutrition group (13).

Data anonymization eliminated the need for informed consent, and all methodologies were in strict accordance with the relevant guidelines and regulations. This retrospective study was approved by the Ethical Review Board of Miyanomori Memorial Hospital, which dispensed with the requirement for informed consent.

### 2.3 Statistical analysis

Continuous variables are presented as mean (SD) or median (IQR), while categorical variables are expressed in the form of percentages (%). To appraise the baseline characteristics, a one-way analysis of variance (ANOVA) was implemented for continuous variables, and a chi-square test was carried out to scrutinize the statistical disparities among the normal nutritional status group, the moderate malnutrition group, and the severe malnutrition group. The correlation between PNI and mortality in patients with dysphagia was investigated using Cox proportional hazards model. Survival curves were constructed using the Kaplan-Meier and log-rank analyses. The likelihood ratio test was employed to probe into the interactions among the subgroups. A two-sided significance level of *P* < 0.05 (two-sided) was taken into account, and all reported *p-values* were < 0.05. All statistical analyses were executed with Free Statistics software version 1.9.

## 3 Results

### 3.1 Baseline characteristics of the study population

Table 1 presents the essential characteristics of the cohort of 248 (97 male and 151 female) patients. The average age of the patients was 83.0 years, with a standard deviation of 9.3. Among them, 180 cases were fed via PEG, and 68 cases were fed via TPN, including 26 cases via PORT, 23 cases via NT-CVC, and 19 cases via PICC. Among the total patient cohort, 86 cases (34.7%) were classified into the severe malnutrition group, 38 cases (15.3%) into the moderate malnutrition group, and 124 cases (50.0%) into the normal nutritional status group. As PNI declined, there was a concomitant increase in the patients' age and a downward tendency in the hemoglobin level (*p* < 0.001). Statistically significant differences were observed among the three groups with respect to cerebrovascular diseases, severe dementia, aspiration pneumonia, PEG, and TPN (*P* < 0.05). In contrast, no remarkable statistical difference was detected in the oral intake recovery (*p* = 0.573).

**Table 1:** Baseline characteristics of patients.

**Variables**	**Total (*n* = 248)**	**1 (*n* = 86)**	**2 (*n* = 38)**	**3 (*n* = 124)**	***p***
Age, Mean ± SD	83.0 ± 9.3	85.6 ± 7.1	84.3 ± 7.4	80.9 ± 10.6	<0.001
**Sex**, ***n*** **(%)**					0.338
Male	97 (39.1)	39 (45.3)	14 (36.8)	44 (35.5)	
Female	151 (60.9)	47 (54.7)	24 (63.2)	80 (64.5)	
CI, *n* (%)	132 (53.2)	35 (40.7)	15 (39.5)	82 (66.1)	<0.001
Dementia, *n* (%)	100 (40.3)	45 (52.3)	23 (60.5)	32 (25.8)	<0.001
Asp, *n* (%)	93 (37.5)	43 (50.0)	14 (36.8)	36 (29.0)	0.009
IHD, *n* (%)	44 (17.7)	21 (24.4)	3 (7.9)	20 (16.1)	0.068
Hemoglobin, Mean ± SD	11.0 ± 2.0	9.8 ± 1.9	11.2 ± 1.7	11.7 ± 1.8	<0.001
Oral, *n* (%)	14 (5.6)	3 (3.5)	2 (5.3)	9 (7.3)	0.573
PEG, *n* (%)	180 (72.6)	48 (55.8)	27 (71.1)	105 (84.7)	<0.001
TPN, *n* (%)	68 (27.4)	38 (44.2)	11 (28.9)	19 (15.3)	<0.001
**Status**, ***n*** **(%)**					<0.001
Death	114 (46.0)	20 (23.3)	17 (44.7)	77 (62.1)	
Alive	134 (54.0)	66 (76.7)	21 (55.3)	47 (37.9)	
PNI, Mean ± SD	37.9 ± 7.5	29.9 ± 4.1	36.7 ± 0.9	43.8 ± 4.4	<0.001

CI, cerebrovascular diseases; Dement, severe dementia; Asp, aspiration pneumonia; IHD, ischemic heart disease; PEG, percutaneous endoscopic gastrostomy; TPN, total parenteral nutrition; Oral, oral intake recovery; PNI, prognostic nutritional index.

### 3.2 Kaplan-Meier curve

**Figure 1:**
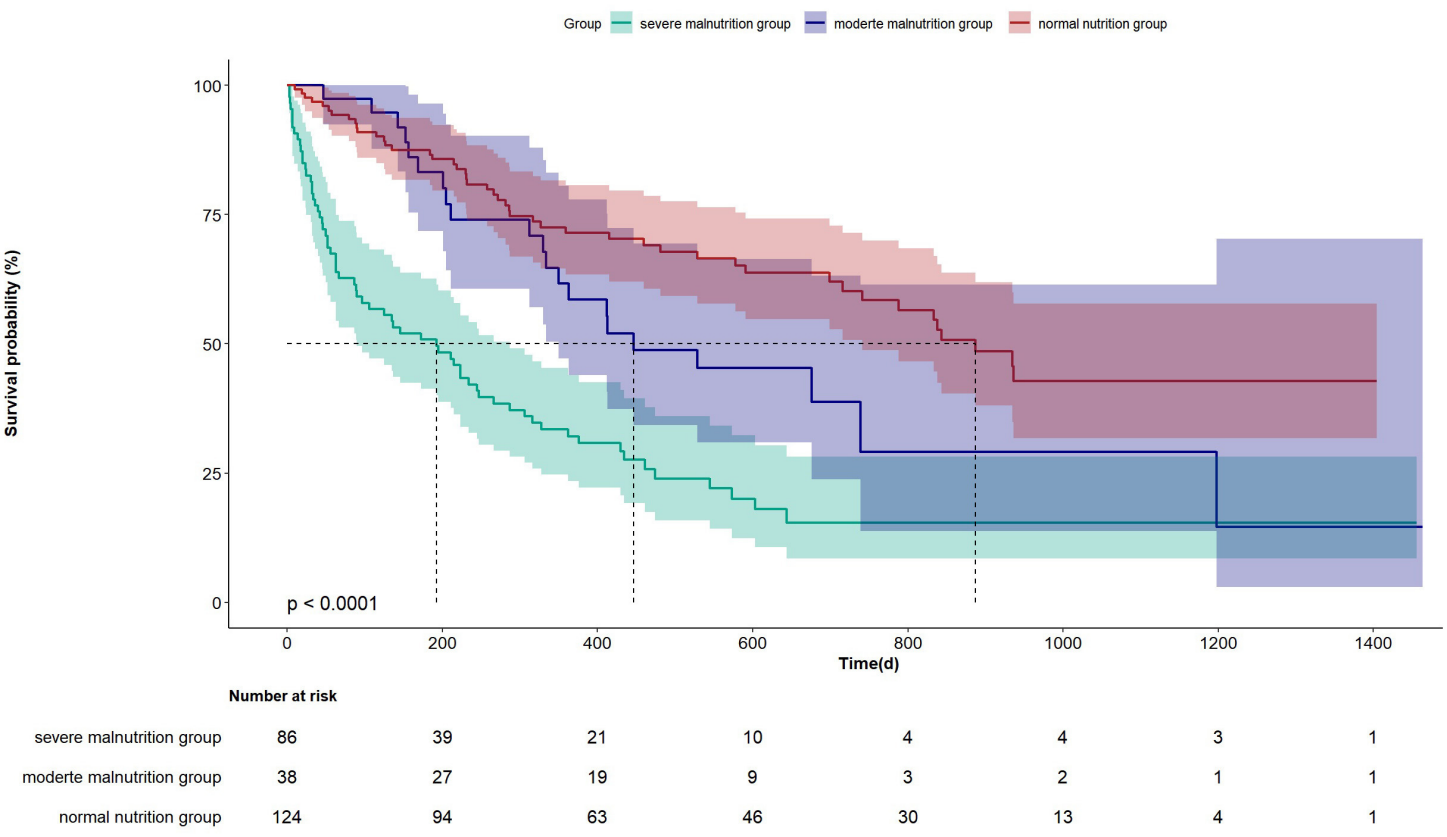
Kaplan-Meier survival analysis for mortality with PNI in three groups.

The Kaplan-Meier curve in Figure 1 indicates that there is a statistical difference in the prognosis among the three groups of patients. The median survival duration of the severe malnutrition group is significantly shorter compared to the other two groups (*P* < 0.0001). The respective values for median survival times were 189, 447, and 864 days for the severe malnutrition group, the moderate malnutrition group, and the normal nutrition status group.

### 3.3 Association between PNI and mortality in various models

Table 2 presents the hazard ratio (HR) and 95% confidence intervals (95% Cl) related to the risk of mortality in patients with dysphagia based on PNI. Four distinct models were formulated, considering PNI as continuous and categorical variables. In model I, no variables were elected for adjustment. For model II, Variables of demographics (age, sex) and past medical history (cerebrovascular diseases, severe dementia, aspiration pneumonia, IHD) were designated for adjustment. In model III, all of the variables (age, sex, IHD, cerebrovascular diseases, severe dementia, aspiration pneumonia, PEG, hemoglobin, TPN, and oral intake recovery) were subjected to adjustment. The results of these models were shown in Table 2. The Univariable Cox regression analysis showed that when PNI was analyzed as a continuous variable, it was negatively correlated with mortality, with a 57% increase in the risk of mortality for every 5-point decrease in PNI (HR = 1.57, 95% CI: 1.39–1.76, *P* < 0.001). Upon adjusting for all covariates in the multivariable Cox regression analysis, the HR was 1.34 (95% CI: 1.16–1.56, *P* < 0.001). Compared to the normal nutrition status group (PNI ≥ 38), the severe malnutrition group (PNI < 35) had a significantly higher mortality, with a statistically significant difference (*P* = 0.007). The adjusted HR values for PNI and mortality in the severe malnutrition group and moderate malnutrition group (35 ≤ PNI < 38) were 1.83 (95% CI: 1.18–2.84, *P* = 0.007) and 1.39 (95% CI: 0.81–2.4, *P* = 0.234), respectively.

**Table 2:** Univariate and multivariate cox proportional hazard model for mortality.

**Variables**	**PNI^a^**		**PNI**				
	**HR (95% CI)**	***P***	**T1 (TNI** < **35)**		**T2 (35** ≤ **TNI** < **38)**		**T3 (TNI** ≥ **38)**
			**HR (95% CI)**	***P***	**HR (95% CI)**	***P***	**Reference**
**Model I**	1.57 (1.39–1.76)	<0.001	3.54 (2.42–5.18)	<0.001	1.66 (0.99–2.78)	0.056	1.0
**Model II**	1.44 (1.27–1.65)	<0.001	2.65 (1.76–3.98)	<0.001	1.4 (0.82–2.4)	0.217	1.0
**Model III**	1.34 (1.16–1.56)	<0.001	1.83 (1.18–2.84)	0.007	1.39 (0.81–2.4)	0.234	1.0

a PNI was entered as a continuous variable per 5U decrease.

Model I: non-adjusted. Model II: adjusted for age, sex, cerebrovascular diseases, severe dementia, aspiration pneumonia, ischemic heart disease. Model III: adjusted for age, sex, cerebrovascular diseases, severe dementia, aspiration pneumonia, ischemic heart disease, total parenteral nutrition, percutaneous endoscopic gastrostomy, oral intake recovery, hemoglobin.

PNI, prognostic nutritional index.

### 3.4 Subgroup analyses

**Figure 2:**
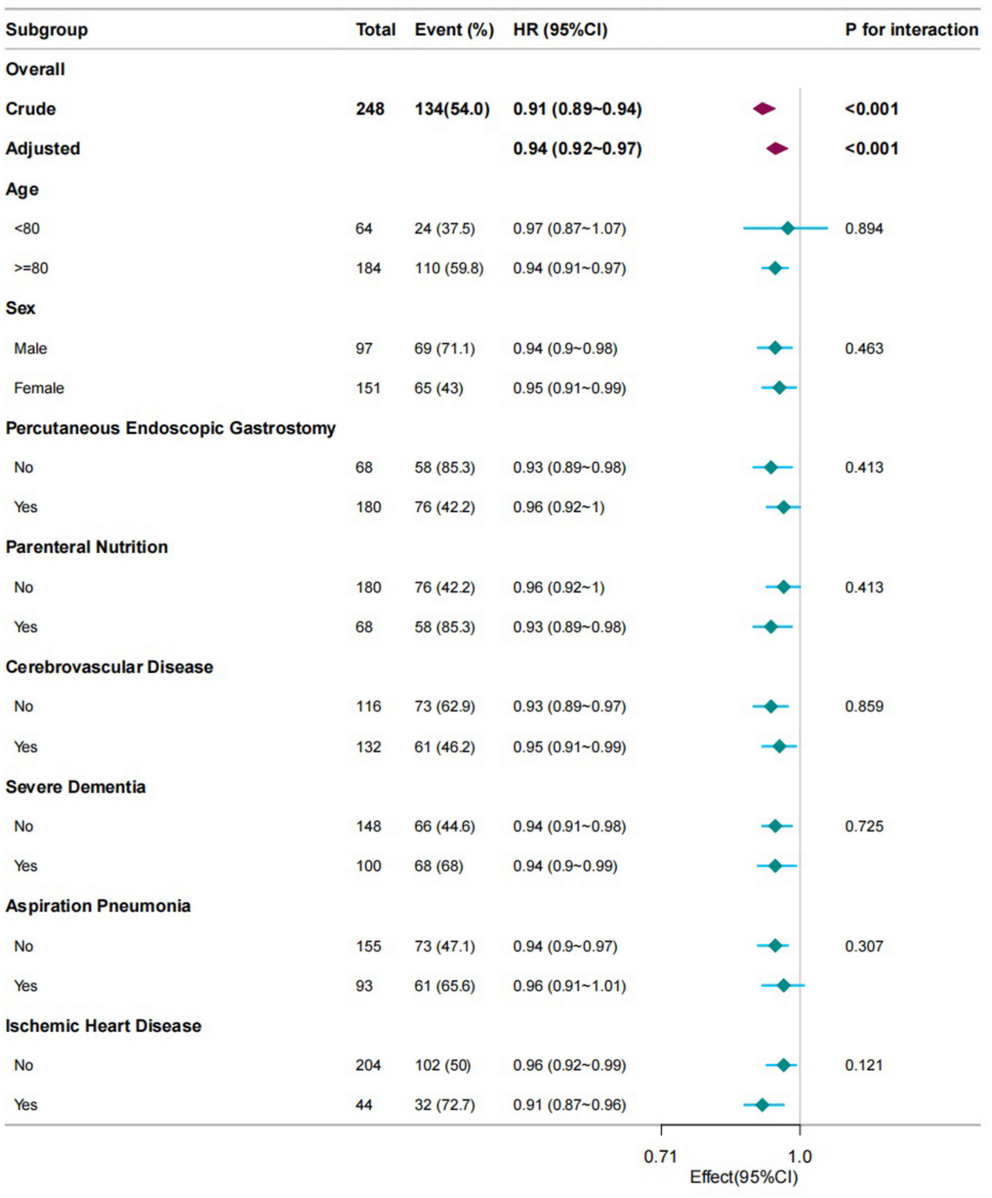
Subgroup analyses of PNI associated with mortality. Hazard ratios (HRs) were adjusted for age, sex, cerebrovascular diseases, severe dementia, aspiration pneumonia, ischemic heart disease, total parenteral nutrition, percutaneous endoscopic gastrostomy, oral intake recovery, and hemoglobin. PNI, prognostic nutritional index.

Subgroup and interaction analyses were conducted to evaluate the consistency of the correlation between PNI and the mortality related to patients with dysphagia (Figure 2). Following the adjustment for all the covariates encompassed in this study, namely age, sex, IHD, cerebrovascular diseases, severe dementia, aspiration pneumonia, PEG, hemoglobin, TPN, and recovery of oral intake, and during the process of subgroup analysis, in case the subgroup analysis variable was a categorical variable, it is excluded from the analysis. No interaction was identified among the eight subgroups. The result was consistent and is depicted in Figure 2.

## 4 Discussion

Dysphagia is a complex process that requires good coordination among multiple tissues and organs. It is regulated and controlled by several regions within the central nervous system and depends on the normal functions of numerous muscles-striated and smooth muscles, in addition to the soft tissues and skeletal anatomy (14). Pathological damage at any point in the swallowing pathway can lead to dysphagia. The three main populations at risk of dysphagia include: people with stroke, patients with neurodegenerative diseases (including Alzheimer's disease, Parkinson's disease, multiple sclerosis, and amyotrophic lateral sclerosis), and patients with head and neck cancers (including oral cancer, nasopharyngeal cancer, and laryngeal cancer) (15, 16). Besides recognizable disease states, the process of healthy aging is also associated with an increased prevalence of dysphagia (15). In the elderly population, existing evidence shows that 9% of care home residents in the Netherlands and 11.4% of community residents in the UK have symptoms of dysphagia (17, 18). Multiple studies in Europe have found that the incidence of dysphagia among community-dwelling elderly is 11.4%18, 17.3% in care homes (19), and 47.4% in hospitals (20). Dysphagia in the elderly can lead to serious complications and has a great impact on patients' health, nutritional status, and quality of life. Developed dysphagia or inefficient nutritional intake leads to malnutrition and/or dehydration, while impaired swallowing safety results in aspiration, respiratory infections, and an increased risk of readmission. All these complications will increase the mortality among this group of people (15, 21–23).

PNI represents a reliable and novel instrument for prognostic assessment, which can assist in predicting patient outcomes in various disease states (6, 24, 25). Our study evaluated the prognosis of elderly patients with dysphagia using PNI calculated based on ALB and total lymphocyte count. It was found that there was a significant negative correlation between PNI and mortality. Specifically, when PNI was analyzed as a continuous variable, for every 5-point decrease in PNI, the mortality increased by 57% (HR = 1.57, 95% CI: 1.39–1.76, *P* < 0.001). Even after adjusting for age, gender, underlying diseases, nutritional intake methods, and hematological parameters, the difference remained statistically significant, with an HR of 1.34 (95% CI: 1.16–1.56) (*P* < 0.001). Further, patients were grouped according to PNI to compare the prognostic differences between the normal nutrition status group and the moderate and severe malnutrition groups. The results showed that in both univariate and multivariate COX regression analyses, PNI was significantly associated with mortality. In the model without adjustment for confounding factors, the mortality of the severe malnutrition group was 3.5 times that of the group with normal nutrition status. After adjusting for all covariates, the multivariate analysis results indicated that the statistical significance of the correlation between PNI and the mortality of dysphagia still existed. The all-cause mortality of the severe malnutrition group was 1.83 times that of the group with normal nutrition status (*p* = 0.007).

PNI was initially used to assess the surgical risks of digestive system tumors (7), and is now being used for prognostic assessment of various tumors, such as early-stage colorectal cancer, renal cancer, cervical cancer, and malignant melanoma (26–28). In recent years, an increasing number of studies have revealed its association with the prognoses of multiple inflammatory diseases. Some scholars have found that among patients with community-acquired pneumonia, a higher PNI is related to a lower mortality (24). Wu et al. (6) investigated the relationship between PNI and all-cause mortality in patients with sepsis and discovered that PNI is an independent risk factor for mortality. In other diseases, including peritoneal dialysis and COVID-19, a decrease in PNI is associated with a poor prognosis (6, 25). Our study has similar findings. For elderly patients with dysphagia, PNI is an independent risk factor for mortality. Particularly in patients with severe malnutrition, the mortality is significantly increased. It is noteworthy that there was no statistically significant difference in mortality between the moderate malnutrition group and the severe malnutrition group as well as the group with normal nutrition status. The possible reason for this might be the relatively small number of cases in the moderate malnutrition group. Further clarification is required with more robust large-scale data studies. However, according to the Kaplan-Meier curve in Figure 1, there was a statistically significant difference in mortality among the three groups (*P* < 0.0001). The median survival duration of the severe malnutrition group, the moderate malnutrition group, and the normal nutrition group were 189 days, 447 days, and 864 days respectively.

Previous studies have found that patients with residual dysphagia after cerebrovascular diseases have a poor prognosis (11, 29), which is related to varying degrees of inflammatory responses (30). The inflammatory process contributes to the mechanisms of injury and repair. Therefore, it is very important to consider the inflammatory state for the prognostic assessment of such patients. PNI combines the level of ALB and the total lymphocyte count. The total lymphocyte count reflects the function of the immune system, and a low level may indicate the presence of immunodeficiency. It has been reported that PNI is associated with inflammatory states such as diabetic nephropathy and infections (31, 32). Wang et al. also found that in young stroke patients, a low PNI was associated with a high level of inflammatory response and was an independent risk factor for poor prognosis at 90 days (33). This correlation emphasizes the potential utility of PNI in studying the inflammatory process and nutritional status of patients with dysphagia. In addition, some scholars have proposed that malnutrition caused by dysphagia will further aggravate the swallowing disorder in patients, creating a vicious cycle, and leading to a poor prognosis (34). Malnutrition restricts the normal function of immune cells, reduces the body's ability to resist infections and regulate inflammatory responses, and ultimately has an adverse impact on the function of the immune system (35). Moreover, malnutrition will promote oxidative stress responses and the production of inflammatory mediators, trigger excessive inflammatory responses, and exacerbate the severity of various diseases (36, 37). Good nutritional status can accelerate recovery, reduce the risk of infection, and improve the overall prognosis of patients with dysphagia. Since the clinical trials by Seltzer et al. (38) ALB has been widely used as a marker of nutritional status. Although the role of ALB in the assessment of nutritional status has been challenged recently, studies have shown that patients with low ALB levels are at risk of malnutrition. One study has determined that ALB < 3.5 g/L is an independent risk factor for predicting a poor prognosis in patients with dysphagia (39). PNI combines the ALB and the total lymphocyte count. These two components, when used together to evaluate the overall nutritional and inflammatory status of patients, have more advantages in predicting patient prognosis compared to a single indicator.

There is a gap in the current research literature regarding the application of PNI to evaluate the prognosis of elderly patients with dysphagia. Our study has found that the mortality of patients with severe malnutrition is significantly increased. Considering this situation, stratifying the nutritional status of elderly patients with dysphagia based on PNI and providing sufficient nutrition to enhance immune function and tissue integrity as well as reduce the incidence of inflammation would prove beneficial for improving the prognosis. The use of nutritional supplements via enteral or intravenous routes constitutes a potential risk factor for infection and may have implications for the outcome (12). Subgroup analyses were conducted separately on patients with PEG and TPN, and the results remained stable. Among patients with dysphagia, regardless of whether nutritional supplements are administered through enteral or intravenous means, PNI serves as an independent risk factor for the mortality. A study focusing on cancer patients revealed that those with a lower PNI already required alternative feeding pathways even in the pre-treatment stage of the tumor (40). Another European study regarding head and neck tumors found that 89.2% of patients with dysphagia and malnutrition were unable to utilize the oral route for nutrition. Research reports indicated that one-fifth of such patients needed enteral feeding or stoma creation. When this option is unavailable, it becomes necessary to provide nutritional support through intravenous or enteral feeding (41). We also carried out subgroup analyses on age, gender, IHD, cerebrovascular disease, severe dementia, and aspiration pneumonia, and the results were all stable with no interaction detected. This suggests that even in the presence of multiple comorbidities, monitoring PNI, and providing adequate nutritional support according to the nutritional status may enhance patient survival. Future research should further refine the exploration of the impact of the dynamic changes of PNI on the mortality.

A true advantage of this study is that ALB and total lymphocyte count are commonly used clinical blood testing indicators, with rapid detection that does not impose additional economic burden on patients. According to our research, this may be an important prognostic indicator and supports wider implementation in the clinical setting. However, it is important to acknowledge the limitations of this study. Firstly, this study was a retrospective study conducted at a single center in Japan, which may introduce bias and inaccuracy in data collection and recording and further robust multi-center prospective studies in different countries are needed for comparison. Secondly, only differences in mortality were evaluated, and other complications were not assessed. Thirdly, in this data, all patients receiving PEG or TPN had clinically evaluated severe dysphagia, while that on alternative enteral nutrition or with milder illnesses were not evaluated. Lastly, this was a secondary analysis of data from a pre-existing database. We did not design or control the data collection methods.

## 5 Conclusion

PNI is negatively correlated with the mortality of elderly patients with dysphagia. The mortality of patients with severe malnutrition is significantly increased. Subgroup analysis showed no interaction, and the results were stable.

## Data Availability

The datasets presented in this study can be found in online repositories. The names of the repository/repositories and accession number(s) can be found in the article/supplementary material.
